# The Spermidine Synthase Gene *SPD1*: A Novel Auxotrophic Marker for *Chlamydomonas reinhardtii* Designed by Enhanced CRISPR/Cas9 Gene Editing

**DOI:** 10.3390/cells11050837

**Published:** 2022-02-28

**Authors:** Robert A. Freudenberg, Luisa Wittemeier, Alexander Einhaus, Thomas Baier, Olaf Kruse

**Affiliations:** Center for Biotechnology (CeBiTec), Faculty of Biology, Bielefeld University, Universitätsstrasse 27, 33615 Bielefeld, Germany; r.freude@uni-bielefeld.de (R.A.F.); luisa.wittemeier@uni-bielefeld.de (L.W.); alexander.einhaus@uni-bielefeld.de (A.E.); thomas.baier@uni-bielefeld.de (T.B.)

**Keywords:** microalgae, CRISPR/Cas9, gene editing, spermidine, *SPD1*, auxotrophy, selectable marker

## Abstract

Biotechnological application of the green microalga *Chlamydomonas reinhardtii* hinges on the availability of selectable markers for effective expression of multiple transgenes. However, biological safety concerns limit the establishment of new antibiotic resistance genes and until today, only a few auxotrophic markers exist for *C. reinhardtii*. The recent improvements in gene editing via CRISPR/Cas allow directed exploration of new endogenous selectable markers. Since editing frequencies remain comparably low, a Cas9-sgRNA ribonucleoprotein (RNP) delivery protocol was strategically optimized by applying nitrogen starvation to the pre-culture, which improved successful gene edits from 10% to 66% after pre-selection. Probing the essential polyamine biosynthesis pathway, the spermidine synthase gene (*SPD1*) is shown to be a potent selectable marker with versatile biotechnological applicability. Very low levels of spermidine (0.75 mg/L) were required to maintain normal mixotrophic and phototrophic growth in newly designed spermidine auxotrophic strains. Complementation of these strains with a synthetic *SPD1* gene was achieved when the mature protein was expressed in the cytosol or targeted to the chloroplast. This work highlights the potential of new selectable markers for biotechnology as well as basic research and proposes an effective pipeline for the identification of new auxotrophies in *C. reinhardtii*.

## 1. Introduction

The green model microalga *Chlamydomonas reinhardtii* [1] has become a powerful biotechnological production host for a wide range of recombinant proteins [2,3,4] and metabolites [5,6,7]. Innovative tools for metabolic engineering, gene editing and synthetic biology are developing rapidly [8,9,10,11,12,13,14]. However, heterologous gene expression requires selection systems to establish sufficient expression levels and to prevent transgene silencing. The number of selectable resistance markers is limited to about 14 antibiotic and drug/ herbicide resistance genes [1] and with increasing biological safety concerns, the demand for alternatives is high [15,16,17]. Auxotrophic markers can not only fill that role, but are also a viable tool for biocontainment [18]. *C. reinhardtii* has been deemed safe for human consumption [19] and genetically modified strains could gain acceptance easier when antibiotic or herbicide resistances are omitted.

CRISPR/Cas-based gene editing in *C. reinhardtii* has advanced steadily over the past eight years, yet reported editing frequencies (percentage of transformants with the desired DNA alteration) vary widely (from 0.45% up to 95%) depending on the applied strain, type of transformation, sgRNA efficiency, Cas enzyme, repair template (donor-DNA) and application of pre-selection [13]. A major milestone for efficient gene editing was the direct transformation with mature Cas9-sgRNA ribonucleoproteins (RNPs), along with the use of a repair template conferring antibiotic resistance or the screening for a visible phenotype [20,21]. Since error-free homologous recombination (HR) is generally rare in *C. reinhardtii* [22,23], most editing attempts rely on error-prone non-homologous end joining (NHEJ) or microhomology mediated end joining (MMEJ). The addition of flanking genomic DNA fragments (hereafter referred to as homology arms) to the repair template is assisting in precise editing [24,25]. Aside from variations caused by the respective target gene, some fundamental features of the CRISPR/Cas9 technology seem to affect editing frequency, e.g., heat shock treatment [26] and the cell cycle state of the pre-culture [27], possibly due to increased DNA accessibility or activated repair mechanisms as stress response.

Since *C. reinhardtii* has a haploid genome during vegetative growth and is routinely cultivated in chemically defined media, auxotrophies are (in principle) easy to establish by (non-)targeted gene knockouts and supplementation of the respective enzyme product. Auxotrophy complementation is usually achieved by transformation of the native gene, which requires a substantial redesign to be employable in modern synthetic biology strategies (e.g., as protein fusion elements). Moreover, the number of established auxotrophic markers is very low in *C. reinhardtii* (i.e., those that sustain normal growth when supplemented) [28]. Reasons for the scarcity of auxotrophies include metabolic and regulatory differences between plants and algae compared to fungi and bacteria, as well as gene redundancies and uptake limitations of metabolites, most importantly regarding amino acids [28,29]. Earliest reports of auxotrophies date back to the 1950s [30], but as of today, only five auxotrophic selection systems are established in *C. reinhardtii* [1,14,28]: Arginine biosynthesis (*ARG9*, *OTC1*, *AGS1*, *ARG7*), the only amino acid auxotrophy, nitrate assimilation (*NIT1/2*), phototrophic growth (acetate dependency; *OEE1, ATPC*) and biosynthesis of the vitamins nicotinamide (*NIC7*) and thiamine (*THI4/10*). The gene responsible for a *p*-aminobenzoic acid auxotrophy [30,31] has not been reported yet, but a recently discovered cobalamin (B_12_, *METE*) auxotrophy could be utilized in the future [32,33].

Polyamine (PA) biosynthesis represents a new and innovative target for auxotrophy exploration, as PAs are ubiquitous in all domains of life including microalgae and are required for cell fitness [34,35,36]. In addition, some of them are secondary metabolites with low cellular titers, thus, disruption of their synthesis could be easy to supplement. Certain PAs have been shown to be taken up by *C. reinhardtii* [37], although it is unclear whether uptake rates can sustain normal growth of respective knockout mutants. In *C. reinhardtii*, native PAs include putrescine (~91 mol%), nor-spermidine (~8 mol%) and spermidine (~1 mol%), with diaminopropane and the structural isomers spermine and thermospermine only detected in extremely low concentrations [38,39,40]. The biosynthesis pathway of nor-spermidine remains elusive [39], but a putative thermospermine synthase gene (*ACL5/SPS1*, Phytozome: Cre06.g251500) was published recently [40] and the genes responsible for putrescine (*ODC1/2*, Phytozome: Cre03.g159500 and Cre16.g683371) and spermidine (*SPD1*, Phytozome: Cre12.g558450) formation are known.

With current opportunities of CRISPR/Cas9-based gene editing and firmly established synthetic biology strategies, a unique opportunity for the exploration of novel and useful auxotrophic markers in *C. reinhardtii* arises. This has proven to be effective in other microalgae like *Phaeodactylum tricornutum* [41].

In this study, the optimization of a CRISPR/Cas9-based gene editing protocol serves as a starting point for an effective pipeline towards the establishment of new auxotrophic selection markers. We demonstrate targeted knockout of *CrSPD1*, leading to a new spermidine auxotrophy, which can be effectively applied in biotechnology and synthetic biology approaches. This is shown by mixotrophic and phototrophic growth characterization of the auxotrophic strains as well as by complementation with several newly designed *SPD1* expression constructs.

## 2. Materials and Methods

### 2.1. C. reinhardtii Cultivation and Nuclear Transformation

*C. reinhardtii* strains UVM4 [42] and the nitrate capable variant N-UVM4 [7] were maintained on solid Tris acetate phosphate (TAP) [43] agar with updated trace element composition [44] containing ammonium or nitrate, respectively, at a continuous photon flux density of 50 µmol/m^2^/s. Spermidine auxotrophic strains were supplemented with 1.45 mg/L spermidine (Sigma-Aldrich Chemie GmbH, Taufkirchen, Germany) if not stated otherwise.

Liquid cultivations were carried out either mixotrophically in TAP or autotrophically in T2P medium [45] with CO_2_-enriched air (3–5% *v*/*v*) and a continuous photon flux density of around 350 µmol/m^2^/s. Spermidine auxotrophic strains in liquid culture were supplemented with 0.75 mg/L spermidine, if not stated otherwise.

Culture synchronization was achieved by use of 12 h light/dark cycles and mixotrophic cultivation in TAP medium for at least seven days. Synchronization was verified by observing cell count throughout the light-phase.

Cell concentration and average diameter were quantified with the Z2 Coulter Counter Analyzer (Beckman Coulter Life Sciences, Krefeld, Germany) according to manufacturer’s instructions. Biomass dry weight (BDW) was determined gravimetrically after centrifugation (5 min at 20,000× *g*) of a 2 mL culture sample and drying the pellet overnight at 105 °C.

Nuclear transformation for ΔSPD1 mutant complementation was achieved via glass bead method [46] using 5 µg of linearized vector DNA and 1.5 × 10^8^ cells in exponential growth phase. Transformants were selected on TAP agar plates with the appropriate antibiotic (10 mg/L paromomycin or hygromycin).

### 2.2. CRISPR/Cas9-Based Gene Editing

Gene editing was based on the direct delivery of pre-assembled Cas9-sgRNA ribonucleoproteins (RNPs) and double stranded donor-DNA (repair template) via electroporation. The protocol presented here is based on previous descriptions [24,25,26,47]. For each target gene, three sgRNA target sites were determined using the online RGEN tools (Cas-Designer) [48]. GC content was limited to 30–70%, the out-of-frame score set above 50 and no mismatches were allowed. The target sites were located in an exon within the first half of the gene and within close proximity of each other to facilitate use of a single pair of homology arms (Supplementary Table S1). All sgRNAs were synthesized using the EnGen^®^ sgRNA Synthesis Kit (New England Biolabs GmbH (NEB), Frankfurt am Main, Germany) following manufacturer’s instructions and digest efficiency was tested in vitro. For this, the target region was amplified from the *C. reinhardtii* genome via PCR and incubated with pre-assembled Cas9-sgRNA RNPs for 30 min at 37 °C. RNPs were denatured by incubation at 80 °C for 10 min and the DNA was separated via agarose gel electrophoresis.

For transformation, a total of 7 × 10^7^ cells were used, which have been kept in the exponential growth phase for at least three days with a harvesting concentration of about 2 × 10^6^ cells/mL. Cells were centrifuged at 1000× *g*, washed by resuspension in 1 mL of TAP-sucrose (40 mM), centrifuged again and resuspended in a final volume of 120 µL of TAP-sucrose followed by subjection to a heat shock at 40 °C for 20 min. RNPs were assembled at room temperature using 8 µg Cas9-NLS (NEB) and 7 µg sgRNA and then combined with 750 ng linearized (BciVI, NEB) repair template (donor-DNA, see Section 2.3).

Electroporation was performed in 2 mm electrode gap cuvettes (Bio-Rad Laboratories GmbH, Feldkirchen, Germany) using a square-wave protocol [49] on the Gene Pulser Xcel System (Bio-Rad Laboratories GmbH) with a voltage of 250 V and a single pulse of 8 ms. After 10 min at room temperature, 1 mL TAP-sucrose was added and the cells were transferred to 6-well microtiter plates containing 4 mL of TAP for recovery at low light (10 µmol photons/m^2^/s) for 24 h. Selection was performed on agar plates containing the appropriate nitrogen source (nitrate or ammonium) and antibiotics (10 mg/L hygromycin) for at least six days at 300 µmol photons/m^2^/s.

Emerging colonies were screened for successful edit of the target gene by phenotype (Section 2.5) and/or colony PCR (cPCR) as described previously [50] using the Q5 High Fidelity Polymerase (NEB). For genomic DNA extraction, the NucleoSpin Microbial DNA kit (Macherey-Nagel GmbH & Co. KG, Düren, Germany) was used according to manufacturer’s recommendations. Primers are available in Supplementary Table S2. PCR products were sequenced by the Sequencing Core Facility (CeBiTec, Bielefeld University, Germany).

### 2.3. Vector Design and Cloning

All plasmids were designed using the standardized modular cloning system (MoClo) [10]. The cloning procedure for creation of repair template (donor-DNA) plasmids for CRISPR/Cas9 editing experiments is visualized in Supplementary Figure S1. Two fragments of genomic DNA (homology arms) of around 500 bp each up- and downstream of the sgRNA target area with a distance between 9 bp and 56 bp from the cut sites were amplified from the *C. reinhardtii* genome via PCR (Q5 High Fidelity polymerase, NEB). The primers (Supplementary Table S2) included overhangs for direct level 1 cloning into positions 1 and 3 and BciVI cut sites for complete removal of vector DNA from the repair template prior to transformation. PCR products were separated via gel electrophoresis, extracted using the peqGOLD Gel extraction Kit (VWR International GmbH, Darmstadt, Germany), digested with BbsI (NEB), purified and ligated into linearized level 1 acceptor plasmids. A hygromycin (*aphVII*) resistance gene, controlled by the PSAD promoter [51] and FDX1 terminator [52], was assembled into a level 1 plasmid for position 2. It was combined with the plasmids containing the homology arms in a final level 2 assembly. In the marker-less repair template plasmids, a short multi-Stop codon insert, carrying a BamHI cut site (Supplementary Table S2), replaced the hygromycin resistance gene.

For complementation of ΔSPD1 strains, the coding sequence (CDS) of the *C. reinhardtii* spermidine synthase SPD1 (Uniprot: A8JGX0) was adapted for high expression capability by codon optimization and insertions of the RBCS2 intron 1 as previously described [53,54], followed by commercial synthesis (GenScript Biotech B.V., Leiden, Netherlands, Supplementary Table S3). The *SPD1* CDS was then cloned into level 1 MoClo vectors with and without C-terminal fusion of the mVenus fluorescence reporter (hereafter YFP, NCBI: AAZ65844) and a terminal Strep-tag II. Expression was driven by the AβSAP(i) [8] or PSAD promoter and FDX1 terminator. For chloroplast targeted expression, the *C. reinhardtii* photosystem I reaction center subunit II (PSAD) chloroplast targeting peptide (CTP) was included and expression controlled by the PSAD promoter as previously described [8]. For comparison, a level 1 vector containing a paromomycin (*aphVIII*) cassette with PSAD promoter and FDX1 terminator was used.

Plasmids were produced as previously described [7]. Briefly, after in vitro assembly they were used to transform chemically competent *E. coli* DH5α cells. Cells were selected on solid LB agar plates containing the appropriate antibiotic (level 0: 50 mg/L streptomycin, level 1: 300 mg/L ampicillin, level 2: 50 mg/L kanamycin), cultivated overnight at 37 °C and the plasmids were extracted using the peqGOLD plasmid isolation kit (VWR), followed by Sanger sequencing (Sequencing Core Facility).

### 2.4. Fluorescence Screening and Imaging

Reporter fluorescence intensity was quantified in liquid culture and on agar plates as previously described [7]. Briefly, an initial pool of colonies was isolated randomly and their fluorescence on the plate level was compared via an in vivo plant imaging system (NightShade LB 985, Berthold Technologies GmbH & Co. KG, Bad Wildbad, Germany) with the filter set for YFP (excitation: 504 nm, emission: 530 nm). To reduce background fluorescence, TAP agar plates were supplemented with 250 mg/L amido black 10B (Carl Roth GmbH + Co. KG, Karlsruhe, Germany) as previously reported [55]. During liquid culture, fluorescence was measured together with optical density at 750 nm in a plate reader (Infinite M200 PRO, Tecan Group Ltd., Männedorf, Switzerland) set up for YFP (excitation: 515/9 nm, emission: 550/20 nm). Untransformed cells were used for signal normalization. Intracellular localization of recombinant proteins was visualized via confocal single cell fluorescence microscopy as previously described [45].

### 2.5. Starch Assay and Calculation of Editing Frequency

After targeting the *STA6* gene with Cas9-RNPs, starch accumulation in the transformant strains was visualized as described previously [55]. Briefly, colonies were spotted onto nitrogen-depleted TAP (TAP-N) agar plates and incubated under continuous light for two days. Sublimated iodine was placed next to the colonies for 10 min. Editing frequency was determined by dividing the number of unstained colonies (green phenotypes) by the total number of colonies screened. Colony PCR (Section 2.2) was used for target genes that did not allow a phenotypical screening. Here, editing frequency was defined as the number of colonies where the PCR product of the target region was shifted in size to match that of the donor-DNA divided by the total number of analyzed colonies.

### 2.6. Polyamine Extraction and Quantification

Polyamines were extracted and quantified as previously described [7]. Briefly, a cultivation sample of 1 mL was centrifuged at 3000× *g* and the cells resuspended in 600 µL of 5% (*v*/*v*) trichloroacetic acid containing diaminohexane (5 mg/L) as internal standard. After brief incubation on ice, the debris was centrifuged at 20,000× *g* and the supernatant used for pre-column ortho-phtalaldehyde derivatization, followed by reversed phase HPLC and fluorescence detection. Representative chromatograms can be found in Supplementary Figure S2.

## 3. Results and Discussion

### 3.1. Nitrogen Starvation of the Pre-Culture Substantially Increases Editing Frequency of CRISPR/Cas9-Based Gene Editing

**Figure 1:**
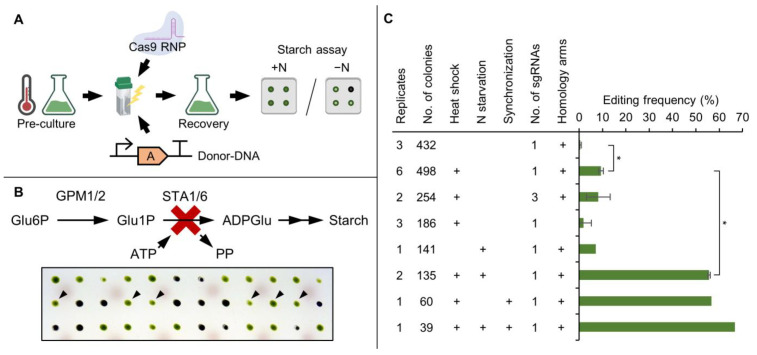
Optimizing Cas9-RNP-based editing frequency using a phenotypical *STA6* knockout screening. (**A**) Transformation experiments started with a specific pre-culture treatment, followed by electroporation of Cas9-sgRNA ribonucleoproteins (RNPs) in combination with a repair template DNA carrying the *aphVII* gene. After 24 h of recovery, cells were plated, selected on antibiotic containing TAP agar plates and emerging colonies were transferred onto plates with and without nitrogen (−N/ +N) for starch assay. (**B**) Pathway of starch synthesis in *C. reinhardtii* and phenotypical screening via starch assay: Functional knockout of the *STA6* gene (red cross) disrupts ADP-glucose formation leading to a starchless phenotype and allowing for visual detection of knockout mutants after exposition to iodine gas (unstained colonies, indicated by black triangles in the second row). (**C**) Effects of the indicated parameters on editing frequency of the *STA6* locus as determined via starch assay. Replicates indicate the number of individual transformations with the total number of colonies screened. Error bars represent the standard deviation of the replicates. Asterisks (*) indicate the significance level of an unpaired, two-sided Student’s *t*-test assuming non-homogenous variances (*: *p <* 0.05). Parts of this figure were created with BioRender.com. (A: Antibiotic resistance, GPM: Phosphoglucomutase, Glu6P: Glucose-6-Phosphate, Glu1P: Glucose-1-Phosphate, STA1/6: Adenosine diphosphate glucose pyrophosphorylase, ADPGlu: Adenosine diphosphate glucose, ATP: Adenosine triphosphate, PP: Pyrophosphate).

Editing frequency determines the success of CRISPR/Cas9-based gene editing, which is routinely monitored via phenotypical changes (such as a light green phenotype for *CpFTSY* [21] or rapamycin resistance for *FKB12* [56]). To determine factors that can lead to increased editing frequencies we coupled pre-selection via an antibiotic resistance marker (repair template) with high-throughput screening of an easy-to-detect starch-less phenotype by targeting the *STA6* gene with Cas9-RNPs (Figure 1A). In this context, editing frequency was defined as the percentage of transformants containing the functional knockout of the target gene due to both HR and NHEJ.

A key step in starch synthesis is the formation of ADP-glucose, which requires catalytic activity of an ADP-glucose pyrophosphorylase containing a small catalytic subunit encoded by the *STA6* gene [57]. The functional knockout of *STA6* consequently results in a starch-less phenotype. Three potential sgRNAs were designed, which were able to guide Cas9 to the correct target site in vitro (Supplementary Figure S3) and sgRNA 3 was used for all subsequent transformations. When targeting *STA6* in vivo, obtained mutants did no longer accumulate starch and were easily distinguishable from non-edited cells on the colony level (Figure 1B). Furthermore, growth on TAP agar plates under mixotrophic conditions was unaffected, demonstrating that there was no bias regarding colony selection after transformation, compared to other phenotypical markers such as *CpFTSY*, where edited colonies grew slower (own observations).

The parameters of the editing assay were optimized stepwise, while using an otherwise standardized set of conditions as control (i.e., heat shock treatment, one sgRNA and use of homology arms). The first parameter was the application of heat shock immediately before transformation (Figure 1C). This was previously determined to be beneficial [26] and let to an increase in editing frequency from 0.5% to about 10% also in this study. Simultaneous application of all three sgRNAs did not significantly improve editing rates, indicating that sgRNAs with target sites in close proximity do not have an additive effect on editing frequency. Omitting the use of homology arms, however, did reduce the editing frequency to about 2%, highlighting their role in double strand break repair.

The molecular implications of heat shock treatment on gene editing have not been characterized yet but we hypothesized that extended application of stress conditions might further increase editing frequencies. To this end, nitrogen (representing a major macronutrient) was removed from the pre-culture 24 h prior to transformation, which increased editing frequencies from 0.5% to 7% without heat shock and from 10% to 55% in combination with heat shock treatment (Figure 1C). This striking effect on editing frequency in both cases makes it likely that nitrogen starvation acts independently of the heat shock treatment.

*C. reinhardtii* does respond quickly to changes in external nitrogen levels. Within hours after removal, a substantial physiological remodeling regarding protein and metabolite composition is triggered. Primarily, nitrogen recycling and uptake capability are increased, leading to a reduction in total protein and RNA contents by 50% and 60%, respectively. Photosynthetic capability is diminished, starch production increased, and the formation of mating competent gametes is induced [58,59]. Furthermore, programmed cell death is triggered in a small fraction of the population [60]. These effects are accompanied by growth arrest, which likely locks cells in G1 phase of the cell cycle. Since the utilization of a synchronized pre-culture [27] also results in increased editing frequencies, a possible explanation for the observed effect might be linked to the cell cycle state.

Indeed, editing frequencies were increased from 10% to 56% when a synchronized culture was transformed at the transition from light- to dark-phase (Figure 1C). A combination of nitrogen starvation and cell synchronization increased editing frequency to 66%. The cumulative effect of both treatments was not as substantial as their individual benefits, which supports the hypothesis of cell cycle arrest being responsible for the increase in editing frequency. Consequently, the optimal conditions were set to nitrogen starvation in combination with heat shock and use of homology arms.

### 3.2. High Throughput Confirmation of Successful Gene Edits by Colony PCR

**Figure 2:**
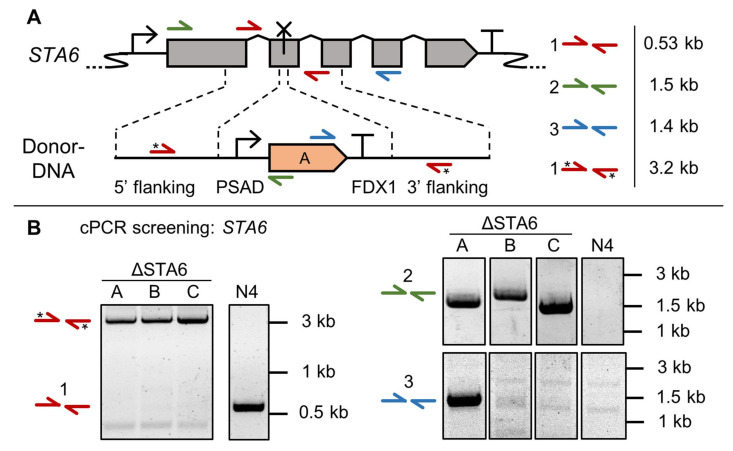
Genotypical verification of ΔSTA6 mutants via colony PCR (cPCR). (**A**) Schematic of the *STA6* gene (not to scale) with sgRNA target site (cross), donor-DNA sequence, homology arms (dashed lines) and primer binding sites (colored half arrows). Asterisks (*) indicate a secondary PCR product derived from amplification of the repair template. Genetic symbols are based on the SBOL 3.0 suggestions or have been published previously [10]. The glyphs for promoter and terminator do include 5‘ and 3‘ UTRs, respectively. (**B**) Representative cPCR results of three independent mutants using the indicated primer pairs. Sequencing results and uncropped gel images are available in Supplementary Figures S4 and S5, respectively. (N4: N-UVM4 parental strain, A: aminoglycoside phosphotransferase *aphVII*, PSAD: Photosystem I reaction center subunit II, FDX1: Ferredoxin).

Colony PCR (cPCR) was performed to attribute the starch-less phenotype to *STA6* target gene disruption. For this, primers were designed to bind in- and outside the homology arms, as well as the *aphVII* repair template (Figure 2A).

When using primer pair 1 (red arrows), 95% of analyzed transformants (63 out of 66) showed a larger PCR product than expected for the intact *STA6* locus. Instead, it correlated with the expected length of the amplified repair template (Figure 2B). The remaining three mutants probably contained small Indels at the sgRNA target site without repair template integration. It is likely that NHEJ at the *STA6* locus and random co-integration of *aphVII* into the genome lead to the desired phenotype but PCR products were not distinguishable from the respective parental PCR product. It can be concluded that this cPCR approach, given its small error rate, offers a fast and reliable confirmation for target gene disruption.

Amplifying from within the *aphVII* CDS towards outside the homology region to confirm targeted repair template integration (Figure 2B, primer pairs 2 and 3) was possible but proved less reliable, i.e., only a few colonies gave rise to a PCR product using primer pair 3. This is likely due to primer binding site deletion after NHEJ. Primer pair 2 on the other hand frequently (6 out of 12) resulted in a PCR product with the expected size for repair template integration via HR, hinting at separately occurring repair events for each homology arm. Sequencing confirmed upstream integration of the repair template by perfect HR in three of the analyzed mutants (Supplementary Figure S4). During amplification of the homology regions (primer pairs 2 or 3), a small risk for miss-amplification remains due to hybridization of separate PCR products [61], so disruption of the target site needs to be confirmed by use of primer pair 1.

### 3.3. SPD1 Is Essential for C. reinhardtii and Its Knockout Creates a Novel Spermidine Auxotrophy

**Figure 3:**
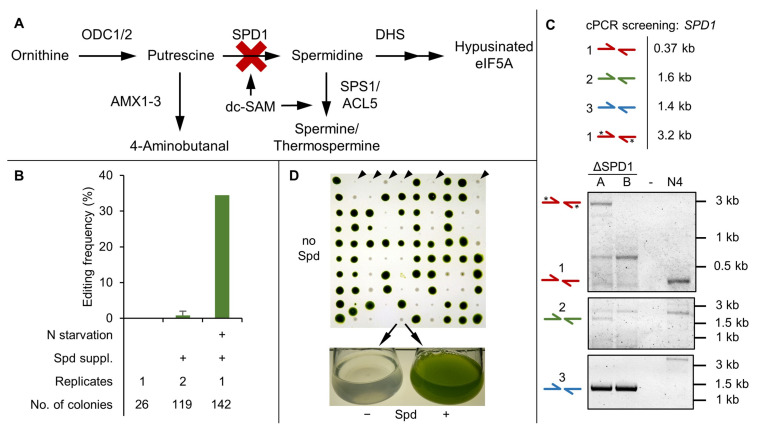
*SPD1* knockout study using Cas9-RNP-based gene editing. (**A**) Schematic of the spermidine metabolic pathway. Functional knockout of the *SPD1* gene (red cross) should disrupt spermidine formation. (**B**) Editing frequency of the *SPD1* locus depending on pre-culture treatment and spermidine supplementation (0.1 mM) as determined by colony PCR and spermidine starvation assay. The number of individual transformations and screened colonies are indicated. Error bars indicate the standard deviation of replicates. (**C**) Representative cPCR results of two independent mutants using the indicated primer pairs (compare Figure 2). Asterisks (*) indicate a secondary PCR product derived from amplification of the repair template. Sequencing results and uncropped gel images are available in Supplementary Figures S6 and S7 respectively. (**D**) Phenotypical screening via spermidine starvation assay. Colonies emerging after transformation were transferred to a spermidine-less plate. Colonies grew slower and bleached after 10 to 14 days, confirming the spermidine auxotrophy of the ΔSPD1 mutants (black triangles in the first row). Colonies were also grown in liquid medium with and without spermidine (lower panel). (N4: N-UVM4 parental strain, ODC: Ornithine decarboxylase, SPD1: Spermidine synthase, DHS: Deoxyhypusine synthase, SPS1: Spermine synthase, ACL5: Thermospermine synthase, AMX: Amine oxidase, dc-SAM: decarboxylated S-adenosylmethionine, eIF5A: eukaryotic translation factor 5A, Spd: Spermidine).

The spermidine synthesis pathway (Figure 3A) in *C. reinhardtii* represents a new promising target for the generation of an auxotrophy by functional knockout of the key enzyme SPD1. Spermidine is an essential molecule for cell cycle progression, the hypusination of the eukaryotic translation (initiation) factor 5A (eIF5A) and spermine synthesis. These processes might be interconnected, as spermine was shown to be causing cell cycle arrest by repressing spermidine formation [62]. Besides that, spermidine could play an important role in abiotic stress tolerance, which has been confirmed for higher PAs in plants [63,64].

For the hypusination of eIF5A, the aminobutyl group of spermidine is covalently bound to a lysine residue of eIF5A catalyzed by the deoxyhypusine synthase (DHS), followed by the action of deoxyhypusine hydroxylase (DOHH) [36,65]. Absence of this hypusination is assumed to be lethal in eukaryotes, because it is essential for protein biosynthesis, not only during translation of polyproline stretches, but also for efficient termination [66]. This has been proposed for *Saccharomyces cerevisiae*, where a spermidine auxotrophy was characterized [67]. Other evidence further suggests that higher PAs are directly involved in translation [68].

The essential nature of *SPD1* in *C. reinhardtii* was confirmed in initial knockout attempts, which did not yield any edited transformants (Figure 3B). However, when supplementing the selection agar plates with 0.1 mM spermidine [62], one out of 119 transformants was found to be edited under non-optimized editing conditions. Our enhanced CRISPR/Cas9 protocol proved highly effective and resulted in an increased editing frequency of 34% (49 out of 142 transformants), which highlights the importance of pre-culture treatment.

Colony PCR using primer pair 1 (Figure 3C) was used for determination of a disrupted target locus, whereas pairs 2 and 3 served as confirmation for targeted insertion of the repair template. However, due to frequent non-homologous end joining (NHEJ) events, amplification was not possible for all the obtained transformants. To assess the frequency of error-free homologous recombination (HR), 36 mutants were analyzed using primers that amplified the entire target region (primers 2 fwd. and 3 rev.). For 15 of these mutants, distinct PCR products were obtained, seven of which were successfully sequenced (Supplementary Figure S6). Only one mutant could be attributed to a scarless edit of the *SPD1* locus by perfect HR of the repair template. In all other mutants at least one side of the repair template was missing and/ or random DNA fragments were additionally integrated as a result of large Indels caused by error-prone NHEJ or imperfect HR. The frequency of NHEJ was also higher upstream of the Cas9 cut site, where the distance between homology arm and cut site was greater (56 bp versus 11 bp).

Maintenance of ΔSPD1 cell lines without spermidine supplementation (Figure 3D) resulted in slowed growth and cell death after 10 to 14 days. The fact that the auxotrophy took longer compared to other auxotrophies (e.g., ammonium depletion in ΔNIT1/2 mutants) to cause a severe phenotype is not only useful for biotechnological application, but also reveals that *C. reinhardtii* requires very little amounts of spermidine. The optimal supplementary spermidine dosage was determined in a spiking experiment involving three ΔSPD1 mutants and the parental strain N-UVM4 with concentrations ranging from 0 to 100 µM (Supplementary Figure S8). A severe reduction in final cell concentration in the auxotrophic strains was observed below 1 µM and above 10 µM spermidine both in liquid and on solid medium. This effect was also observed for the parental strain, hinting at previously unknown cytostatic effects of high spermidine concentrations. Therefore, a spermidine dosage of 5 µM (0.75 mg/L) in liquid culture and 10 µM (1.45 mg/L) for agar plates (due to long-term instability of spermidine [69]) should be applied. This dosage is relatively low and can be compared to the demand for the cofactor manganese (6 µM in TAP medium [44]).

### 3.4. Complementation of the ΔSPD1 Mutant Has Versatile Biotechnological Utility

**Figure 4:**
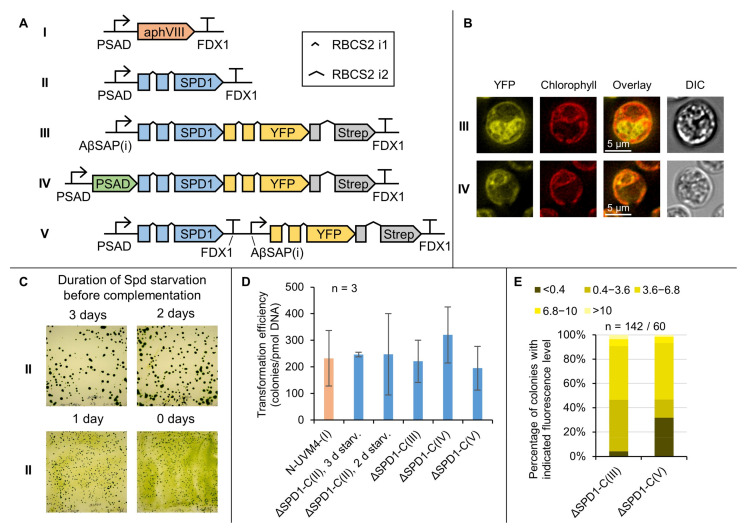
Complementation of the ΔSPD1 mutation. (**A**) Genetic constructs created for the experiment. The *SPD1* gene from *C. reinhardtii* was codon optimized and equipped with introns. The PSAD chloroplast targeting peptide is highlighted in green. The number of introns is correctly represented, their placement is not to scale. Genetic symbols are based on the SBOL 3.0 suggestions or have been published previously [10]. The glyphs for promoter and terminator do include 5‘ and 3‘ UTRs, respectively. (**B**) Representative single cell fluorescence microscopy pictures of ΔSPD1 mutants complemented with constructs III and IV to confirm subcellular localization of the SPD1-YFP fusion protein. (**C**) Effect of spermidine starvation prior to complementation of the ΔSPD1 mutation. Cells were transferred to spermidine-less medium and kept in the exponential growth phase for the indicated number of days prior to transformation with construct II. Pictures were taken seven days after plating. (**D**) Transformation efficiency depending on the genetic construct. Three independent ΔSPD1 mutants were complemented with the indicated construct. Error bars represent standard deviation. (**E**) *SPD1* expression levels in ΔSPD1 mutants complemented with constructs III and V as indicated by YFP fluorescence level. Signal intensities of <0.4 and >10 are outside of detection limits. (PSAD: photosystem I reaction center subunit II, FDX1: ferredoxin, RBCS2 i1/i2: ribulose bisphosphate carboxylase small subunit intron 1/2, AβSAP(i): HSP70A/βTUB2 Synthetic Algae Promoter, SPD1: spermidine synthase, *aphVIII*: aminoglycoside 3’-phosphotransferase type VIII, DIC: differential interference contrast, Spd: spermidine, ΔSPD1-C(n): SPD1 null mutant complemented with indicated construct number).

An optimized SPD1 CDS was used for complementation of ΔSPD1 mutants. Four expression vectors were designed (Figure 4A) to determine native SPD1 localization (as no signal peptide could be predicted using PredAlgo 1.0 [70]) as well as its potential application as a fusion partner for expression in the cytosol and post-translational import into the chloroplast.

Single cell fluorescence microscopy was conducted (Figure 4B) to reveal that the native localization of the SPD1 enzyme appears to be the cytosol and that targeted expression to the chloroplast is indeed occurring when applying the PSAD targeting peptide.

Due to the comparably slow selection process upon spermidine deprivation, cells needed to be pre-starved for spermidine for at least two days prior to complementation, during which they were kept in the exponential growth phase (Figure 4C and Supplementary Figure S9). This prevented severe biomass accumulation on the selection plates. Constructs I to V were used for the transformation of three independent ΔSPD1 mutants and resulting transformation efficiencies were compared to the application of an antibiotic selection system (*aphVIII*, Figure 4D). SPD1 complementation resulted in similar transformation efficiencies compared to commonly used paromomycin selection, confirming appropriate expression of all construct variants. Interestingly, complementation was successful when SPD1 either remained in the cytosol after translation or was post-translationally imported into the chloroplast (constructs III and IV, Figure 4A,B). This may be beneficial for biotechnological applications, in which SPD1 can serve as a fusion partner in advanced genetic engineering strategies. It also suggests that spermidine can pass the chloroplast membrane in sufficient amounts, which is in line with the low spermidine requirements.

Expression levels were compared between constructs III and V (Figure 4E) to see the effect of a translational fusion of YFP to the C-terminus of SPD1. With construct V, about 30% of transformants exhibited YFP reporter fluorescence below the detection limit, indicating a high probability for reporter gene silencing or partial DNA integration. With construct III, however, nearly all colonies exhibited reporter fluorescence above detection limit, which means that a certain level of transgene expression is assured and offers the possibility to omit an additional reporter fusion.

**Figure 5:**
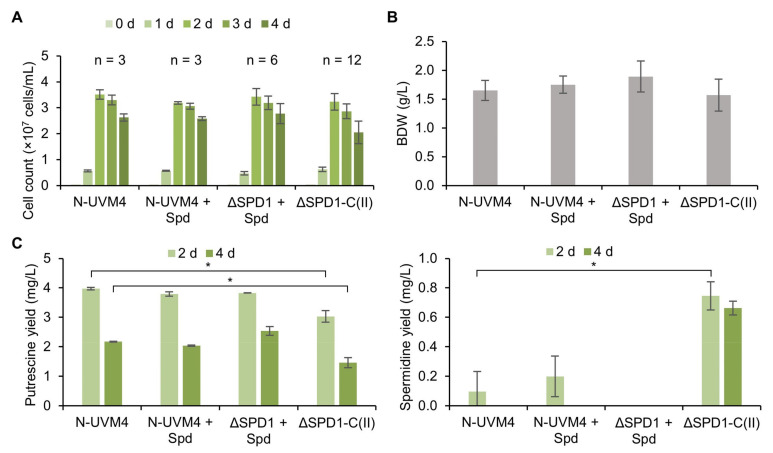
Mixotrophic growth performance of ΔSPD1 mutant strains compared to complemented strains and strain N-UVM4. Three *SPD1* knockout strains were complemented using construct II, of which four progenitor strains were cultivated each. (**A**) Cell concentration over the course of the cultivation. (**B**) Biomass dry weight at day four. (**C**) Volumetric putrescine (left) and spermidine (right) yields of the cellular fraction at days two and four. For missing columns, yields were below the limit of detection. Error bars represent the standard deviation of indicated biological replicates. Asterisks (*) indicate the significance level of an unpaired, two-sided Student’s *t*-test assuming non-homogenous variances (*: *p <* 0.05). (Spd: spermidine, ΔSPD1-C(n): *SPD1* null mutant complemented with indicated construct number).

ΔSPD1 mutant strains were cultivated alongside their complemented offspring and parental strain N-UVM4. Mixotrophic growth (Figure 5A,B) was comparable in all strains regarding cell count and biomass accumulation. Putrescine levels in the complemented strains (Figure 5C) were slightly reduced by 23.7% to 3.03 mg/L and 32.5% to 1.47 mg/L after two and four days, respectively, likely due to elevated expression of the optimized *SPD1* gene. This was confirmed by an increase in spermidine accumulation in these strains (Figure 5C).

Comparable growth performance was also observed when cultivating these strains under phototrophic conditions (Supplementary Figure S10). Here, biomass accumulation was unaffected, but cell counts were slightly reduced for the ΔSPD1 mutant strains and their complemented offspring. Putrescine accumulation on days two and four was highest in the ΔSPD1 mutant strains, indicating that putrescine levels are partially regulated by SPD1 activity in these conditions. Spermidine levels in the complemented strains were comparable to those under mixotrophic growth conditions.

These results indicate that complementation and subsequently elevated spermidine levels (due to SPD1 overexpression) have very little impact on growth in *C. reinhardtii*. Phototrophic growth is slightly affected, hinting at a possible involvement of spermidine in photosynthesis. It has been reported that higher PAs are assisting in direct and indirect protection of the photosynthetic apparatus in plants [71].

To compare strains with particularly high or low expression of SPD1, construct III (Figure 4A) was used and the cells were selected based on fluorescence level (Supplementary Figure S11). With a dynamic expression range of about two orders of magnitude (89-fold difference in reporter fluorescence), no significant difference in growth or biomass accumulation was observed, while spermidine levels differed only by a factor of 4.4 (0.3 mg/L versus 1.3 mg/L). Cellular putrescine levels were unaffected. This highlights the robustness of the complementation with the optimized *SPD1* construct. It also appears that spermidine levels in the cell are tightly regulated by degrading enzymes (likely a polyamine oxidase, PAO [34]) or that the catalytic activity of *Cr*SPD1 is generally slow, given the small difference between low and high expressing strains.

### 3.5. A Marker-Less ΔSPD1 Mutant Strain

**Figure 6:**
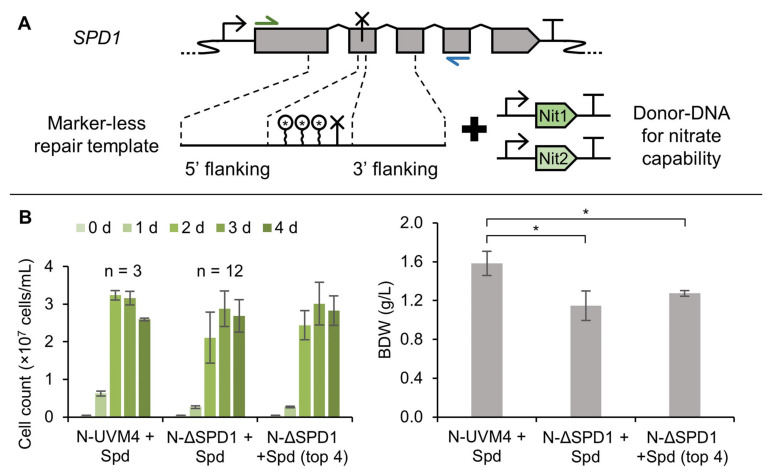
Generation of a markerless *SPD1* knockout strain using nitrate capability for pre-selection. (**A**) The *SPD1* locus was edited in the strain UVM4 using a marker-less donor-DNA containing stop codons and a BamHI restriction site together with the plasmids pMN24 (*NIT1*) and pMN68 (*NIT2*) [72,73] for pre-selection on nitrate containing agar plates. (**B**) Growth performance of ΔSPD1 mutant strains in nitrate containing TAP medium compared to the N-UVM4 strain. Cell concentration over the course of the cultivation is shown (left) as well as biomass dry weight at day four (right). The four best performing strains regarding biomass formation are also shown separately. Error bars represent the standard deviation of indicated biological replicates. Asterisks (*) indicate the significance level of an unpaired, two-sided Student’s *t*-test assuming non-homogenous variances (*: *p <* 0.05).

High editing frequencies still rely on pre-selection after transformation and it was not possible to obtain a ΔSPD1 mutant strain solely with a marker-less repair template due to low editing efficiencies in this study. Therefore, the nitrate auxotrophic strain UVM4 was used for co-transformation with a marker-less repair template for the *SPD1* locus together with the genes for nitrate capability (*NIT1* and *NIT2*) and selection on nitrate containing medium (Figure 6A). Using the optimized CRISPR/Cas9 protocol, 16 nitrate capable ΔSPD1 mutant strains were obtained and characterized by colony PCR. None of these strains were created by perfect HR with the marker-less repair template, which highlights the low probability of HR in *C. reinhardtii*. Growth was compared to the nitrate capable strain N-UVM4 (Figure 6B) and showed some variation among the mutant strains. This can be explained by improperly tuned expression of *NIT1* and/or *NIT2* and strain-to-strain variations. While the best performing strains did not differ significantly from the equivalent strain N-UVM4 regarding cell count, biomass accumulation was reduced by 20%. Nevertheless, targeted and marker-less editing of the *SPD1* gene was possible. The resulting strain could serve as a starting point for further characterization of the spermidine auxotrophy and our work offers a sophisticated pipeline for the establishment of new selectable markers in *C. reinhardtii*.

## 4. Conclusions

With this work, we advance precise gene editing in *C. reinhardtii* and provide new insights into the cellular physiology and importance of the spermidine synthase (SPD1) and its product spermidine.

Starvation of pre-cultures for nitrogen raises editing frequencies and is a valuable addition to the repertoire of optimizations that have been implemented for CRISPR/Cas9-based gene editing approaches in *C. reinhardtii*.

The *SPD1* knockout and resulting spermidine auxotrophy has been demonstrated to be an excellent, novel selection marker for *C. reinhardtii*. Supplementation is cheap, easy to facilitate and does not interfere with growth under investigated conditions. The auxotrophy not only offers a valid biocontainment strategy, but can also be utilized for biotechnological applications, as complementation with a synthetic *SPD1* gene is versatile and robust.

## Data Availability

All data generated in this study are available in this published article and Supplementary Materials.

## Supplementary Materials

The following supporting information can be downloaded at: https://www.mdpi.com/article/10.3390/cells11050837/s1, Table S1: List of sgRNA target sites, Table S2: List of Primers, Table S3: Sequence of the synthetic SPD1 CDS, Figure S1: Cloning procedure for repair template vectors, Figure S2: Chromatograms for polyamine quantification, Figure S3: In vitro sgRNA digest, Figure S4: Sequencing results of the *STA6* locus after editing, Figure S5: Agarose gels after cPCR of the ΔSTA6 mutants, Figure S6: Sequencing results of the *SPD1* locus after editing, Figure S7: Agarose gels after cPCR of the ΔSPD1 mutants, Figure S8: Spermidine supplementation requirements of the ΔSPD1 mutant, Figure S9: Effect of spermidine starvation prior to complementation, Figure S10: Autotrophic growth of ΔSPD1 mutants, Figure S11: Mixotrophic growth of strains with low and high levels of SPD1-YFP expression.
