# Supplementary material for: The Spermidine Synthase Gene SPD1: A Novel Auxotrophic Marker for Chlamydomonas reinhardtii Designed by Enhanced CRISPR/Cas9 Gene Editing
Source: Cells. 2022 Feb 28;11(5):837. doi: 10.3390/cells11050837 (PMC8909627; doi:10.3390/cells11050837)
Supplement: Supplementary file 1 [file cells-11-00837-s001.zip › cells-1575483-supplementary.pdf]

Table S1: Table with all tested sgRNA target sites (including PAM), of which the ones used for transformation are highlighted with bold letters.

Gene	Phytozome identifier	Target exon	Target site (5'-3')	GC content (%)
STA6	Cre03.g188250	4	CAGGCGATAGTTGGCGCCCAGGG	65
			TTGGCACGCTTCTTGGTCAGAGG	55
			**CAAGAAGCGTGCCAAGCCGGCGG**	65
SPD1	Cre12.g558450	2	TCGCCAATGTGGCCTGGGCAGGG	65
			**TCTCCTTAACTTTCAGGGAGAGG**	45
			CACAGGCGATCAACGATGGCTGG	60

Table S2: Table with the most important primers that were used during this study. Capitalized letters indicate BbsI recognition and cut sites as well as BciVI recognition sites.

Name	Use	Sequence (5'-3')
P102_Sta6_E4_H1_F	Amplification of homology arms	attaGAAGACatTGCCGTATCCctgaagatgcgggtgagccag
P103_Sta6_E4_H1_R		attaGAAGACatTTGCcagacgggtgccggcaccac
P104_Sta6_E4_H2_F		attaGAAGACatACTAtcgatattcccgttagcaactgcc
P105_Sta6_E4_H2_R		attaGAAGACatGTAAGTATCCtgtagccgcccacagacgag
P129_SPD1_E2_H1_F		attaGAAGACatTGCCGTATCCaaatgctcaagtcgccgacc
P130_SPD1_E2_H1_R		attaGAAGACatTTGCcgcctgtgggatcagtcagc
P131_SPD1_E2_H2_F		attaGAAGACatACTAggagattctgtaccggggca
P132_SPD1_E2_H2_R		attaGAAGACatGTAAGTATCCgccgtgctgctgcagaggc
P93_HygR_F	Sequencing/ PCR	atccggaggaactggcgcagttc
P94_HygR_R		gcaggctcgcgtaggaatcatcc
P95_FDX_F		tgacacggtgacacgcaggtatacg
P96_P-PSAD_R		ctgacgagggctcgtgtgacg
P133_SPD1_E2_S1_F		ctagggtgtatgcactccgg
P134_SPD1_E2_S1_R		gaggaaagggccaggcttgg
P167_SPD1_E2_N1_F		gctccgctactagtgctcgcc
P168_SPD1_E2_N1_R		ccacggcagccatggcttgc
P63_Sta6_3_F		gttcgggctcaggctgtgtcgacc
P86_Sta6_5_R		tggttcgctcctgtgccctagc
P163_Sta6_E4_N1_F		tggggttcacggtacagcaggc
P164_Sta6_E4_N2_R		gctcttgttggcggacgactgg
P92_Sta6_5_R		Cctcctcgaacagccacatgtactggc
P298_STOP_p2_F	Multi-Stop insert	attaGAAGACatgcaatgactgactagatggatccgtgcatttactaatGTCTTCatta
P299_STOP_p2_R		taatGAAGACattagtaaatgcacggatccatctagtcagtcattgcatGTCTTCtaat

Table S3: Sequence of the synthetic *Cr*SPD1 coding sequence used in this study for complementing the ΔSPD1 mutant. 5′ and 3′ extensions (lower case letters) have been added to facilitate cloning into a MoClo level 0 acceptor vector for position B3. The RBCS2 introns are presented in lower case letters.

**Synthetic *Cr*SPD1 for B3 (1265 bp)**
gaagacaaaatgGCCAGCAAGCCCAGCCCCGGCAGCGCCGACCAGGCCATCAACGACGGCTGGTACACCGAGCTGAGCCC<br>CATGTGGCCCGGCCAGGGCCTGAGCCTGAAGGTGAAGGAGATCCTGTACCGCGGCAAGAGCGACTTCCAGGACGTGTGC<br>GTGTTCGAGAGCGAGAGCATGGGCACCGTGCTGCTGCTGGACGGCGTGATCCAGGCCACCGACCGCGACGAGTTCAGCT<br>ACCAGgtgagtcgacgagcaagcccggcggatcaggcagcgtgcttgcagatttgacttgcaacgcccgcattgtgtcgacgaaggcttttggctcctctgtcgctgtctcaagca<br>gcatctaaccctgcgtcgccgtttccatttgcagGAGATGATCGCCCACATCCCCATGTGCGCCCTGGAGCGCCCCGCCAAGAAGGTGCTG<br>GTGGTGGGCGGCGGCGACGGCGGCGTGCTGCGCGAGCTGGCCCGCTACCCCGACGTGGAGGAGATCCACATGGCCGA<br>GATCGACAAGATGGTGCCCGACGTGAGCAAGCAGTATTTCCCCGAGATGGCCGTGGGCTTCAGCGACCCCCGCGTGACC<br>CTGCACATCTGCGACGGCATCAAGTACGTGGAGGACAGCCCCGAGCACAGCTACGACCTGATCGTGGTGGACAGCAGCG<br>ACCCCGTGGGCCCCGCCGAGGTGCTGTTCGAGAAGCCCTTCTTCGAGGCCCTGCACCGCGCCGTGCGCCCCGGCGGCA<br>TCGTGTGCACCCAGGCCGAGAGCCTGTGGCTGCACCTGGACATCATCAAGGCCCTGGCCGGCATGTGCAAGGAGGTGTT<br>CGCCGGCGGCAGCGTGAGCTACGCCACCACCACCATCCCCACCTACCCCAGCGGCCAGATCGGCATGCTGGTGTGCGCC<br>AAGgtgagtcgacgagcaagcccggcggatcaggcagcgtgcttgcagatttgacttgcaacgcccgcattgtgtcgacgaaggcttttggctcctctgtcgctgtctcaagcagca<br>tctaaccctgcgtcgccgtttccatttgcagGCCCGCACCGAGCAGGGCGAGGGCGGCAGCGCCCCCCTGGACCCCCGCATCCCCCG<br>CCAGGCCGAGCCCGGCCCCCTGCCCCAGCTGGGCGTGCCCGAGCTGAAGTATTACAGCCACGAGGTGCACACCGCCGC<br>CTTCGTGCTGCCCGTGTTCGCCAAGAAGGGCCTGGAGGGCTGCCTGACCTTCCAGaggtttgtcttc

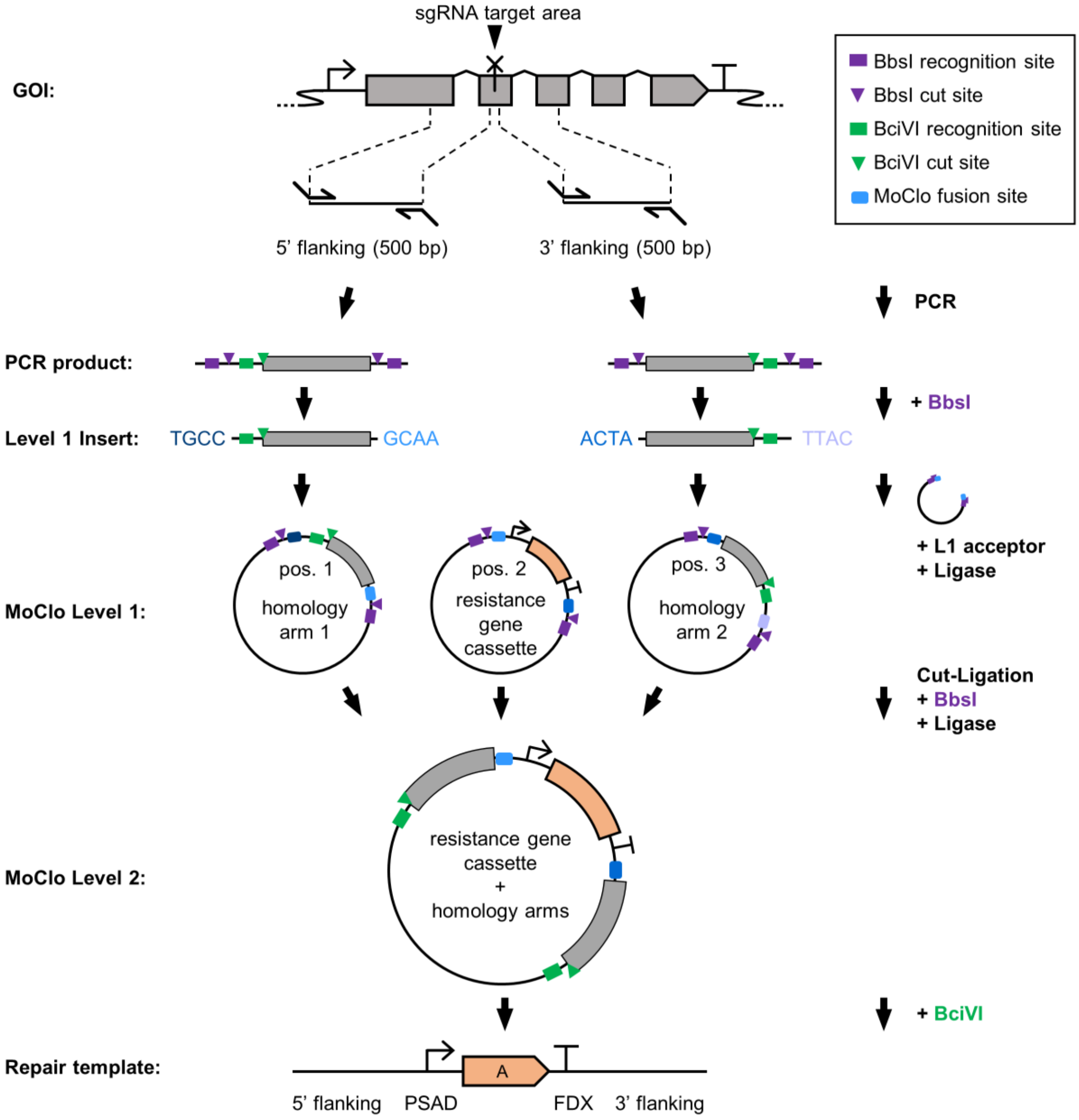

Figure S1: Cloning procedure for the repair template vectors using the MoClo system. The antibiotic resistance gene cassette can be exchanged for any other position 2 insert.

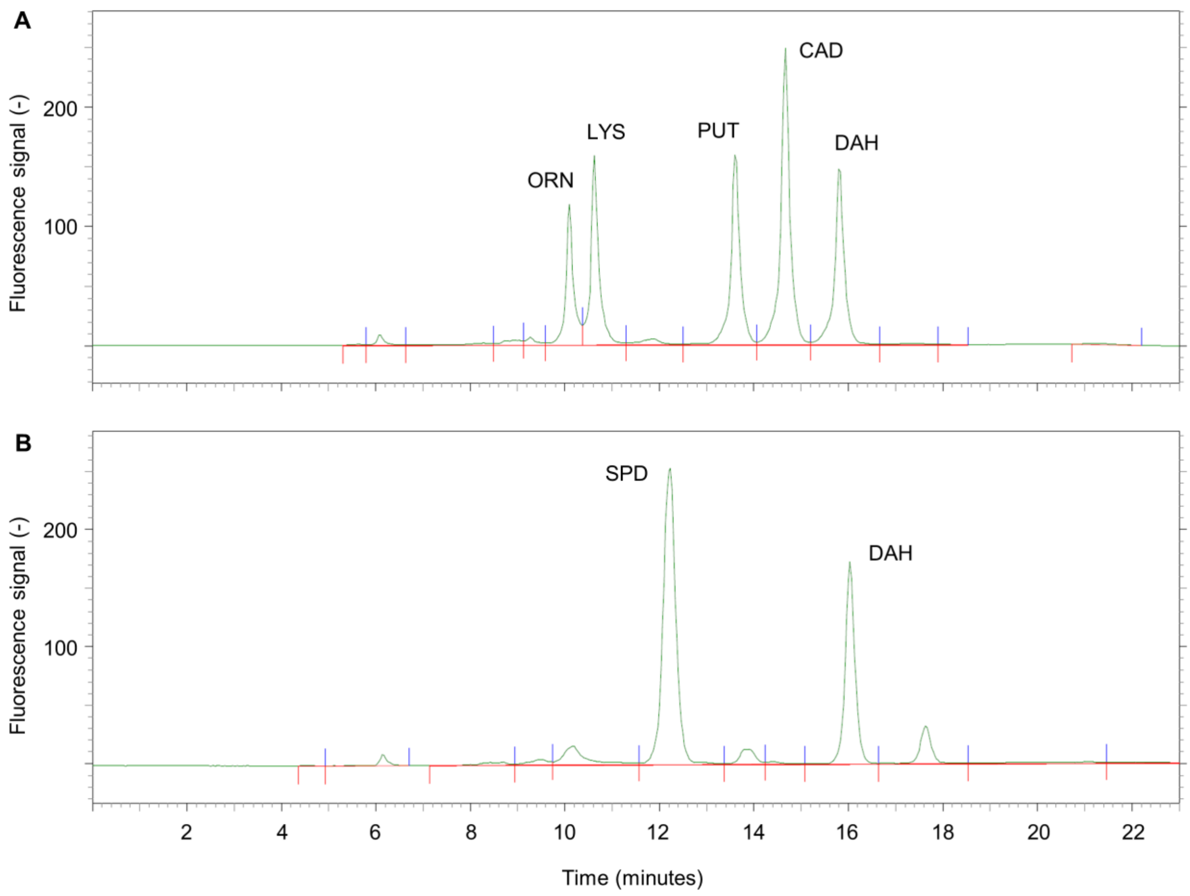

Figure S2: Typical fluorescence chromatograms for polyamine quantification after RP-HPLC and pre-column OPA derivatization. (**A**) Separation of 10 mg/L of each of the following compounds: Ornithine (ORN), lysine (LYS), putrescine (PUT), cadaverine (CAD) and the internal standard diaminohexane (DAH) using 5 mg/L. (**B**) Separation of 150 mg/L of spermidine (SPD) and the internal standard DAH.

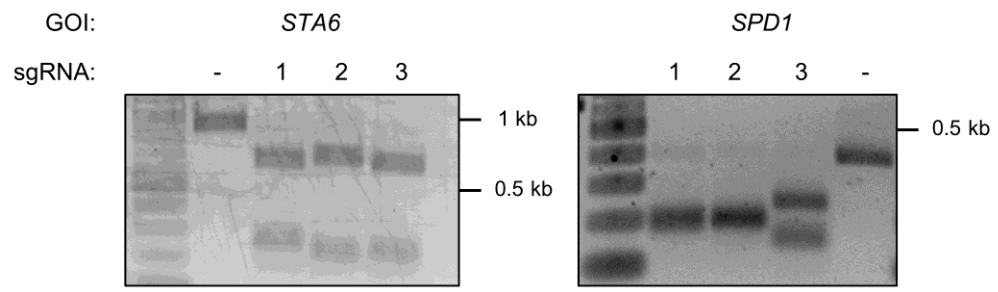

Figure S3: Agarose gel after *in vitro* digest of the sgRNA target region for *STA6* and *SPD1* using three different Cas9-sgRNA RNPs each (see Supplemental table S1).

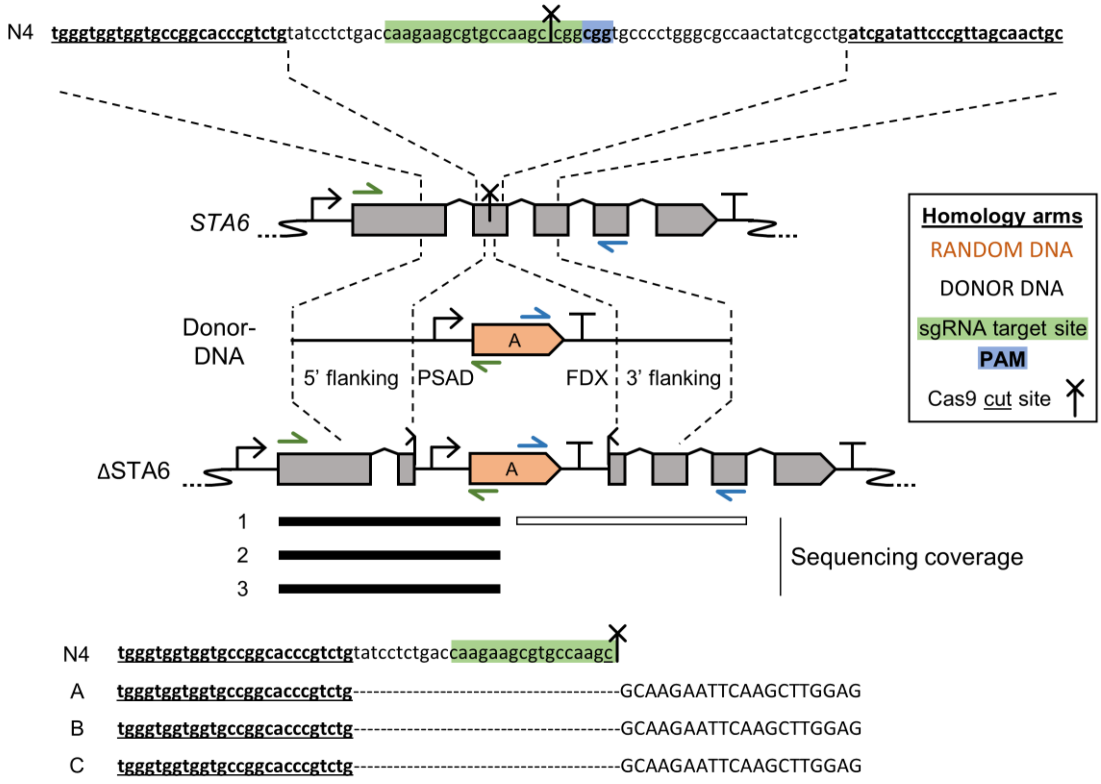

Figure S4: Sequencing results of the *STA6* locus after successful Cas9 edit for three different mutants. A hygromycin resistance cassette with homology arms was used as repair template. The genetic layout of the parental strain N-UVM4 is presented at the top, both in detail and as schematic (not to scale). Primers used for PCR amplification are color coded. Results are available for the green primer pair. The *STA6* locus edited via homologous recombination is depicted as well as sequencing coverage of the three PCR products. For the sgRNA target region, the sequencing results are displayed in detail at the bottom.

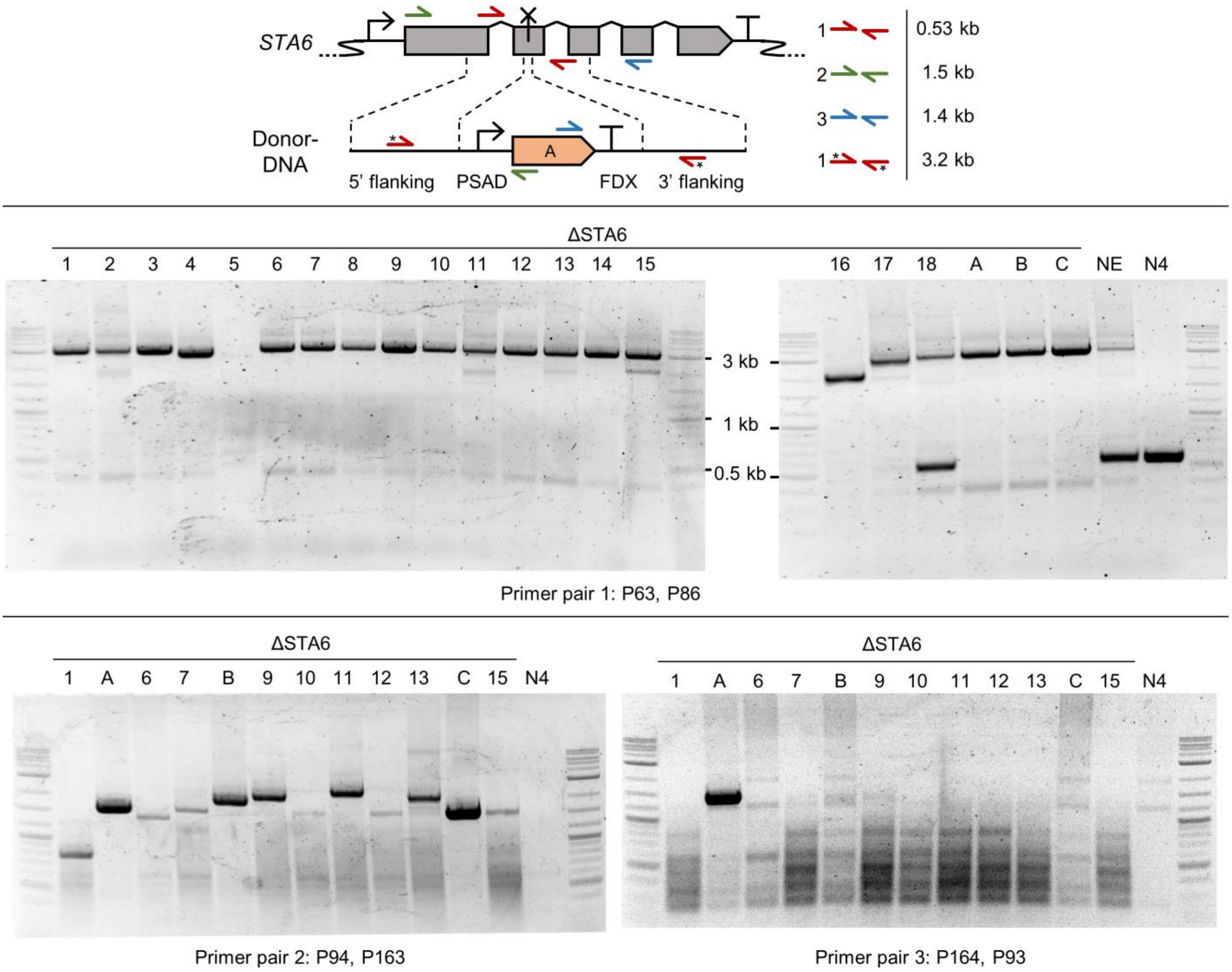

Figure S5: Agarose gels after cPCR of ΔSTA6 mutants using indicated primers. Asterisks (*) indicate a secondary PCR product derived from amplification of the repair template. (N4: N-UVM4 parental strain, NE: non-edited mutant strain with hygromycin resistance)

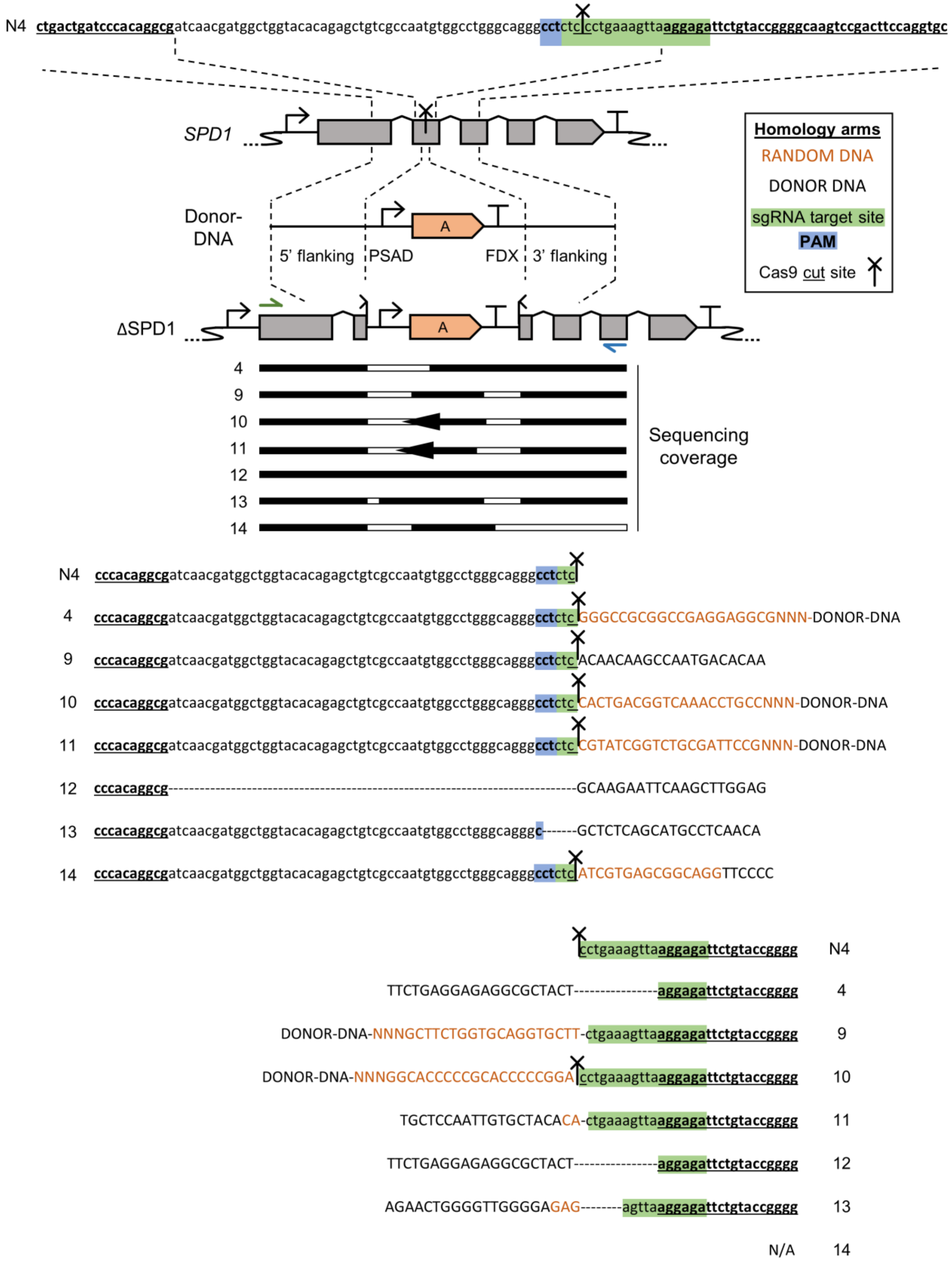

Figure S6: Sequencing results of the *SPD1* locus after successful Cas9 edit for seven different mutants. A hygromycin resistance cassette with homology arms was used as donor-DNA. Top to bottom: The genetic layout of the parental strain N-UVM4 is presented at the top, both in detail around the sgRNA target site and as schematic (not to scale). Primers used for PCR amplification are color coded. Results are available for the green and blue primer combination. The *SPD1* locus edited via homologous recombination is depicted as well as sequencing coverage of the three PCR products. Arrows indicate a reverse orientation of the repair template. For the sgRNA target region, the sequencing results are displayed in detail at the bottom.

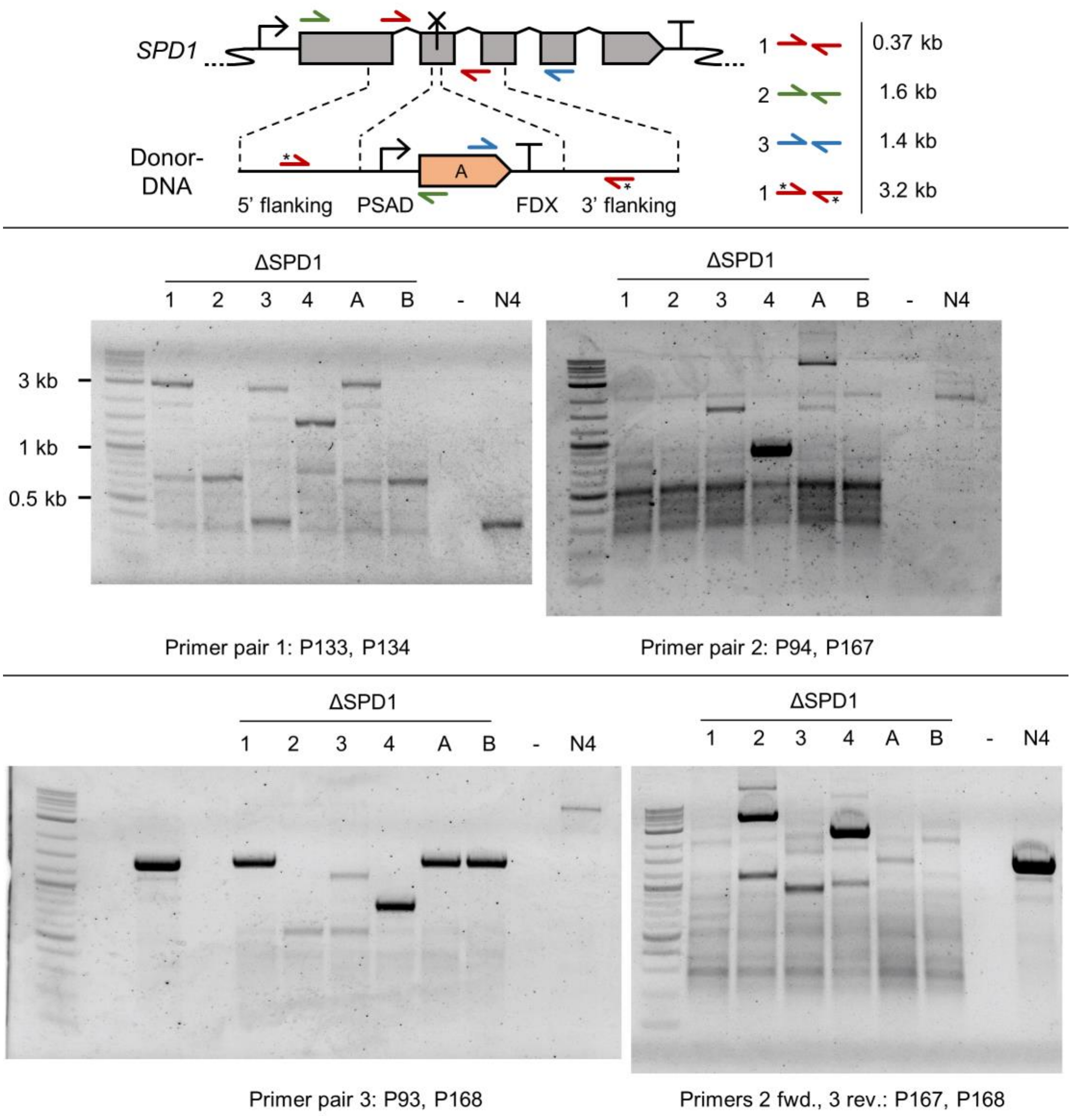

Figure S7: Agarose gels after cPCR of ΔSPD1 mutants using indicated primers Asterisks (*) indicate a secondary PCR product derived from amplification of the repair template. (N4: N-UVM4 parental strain)

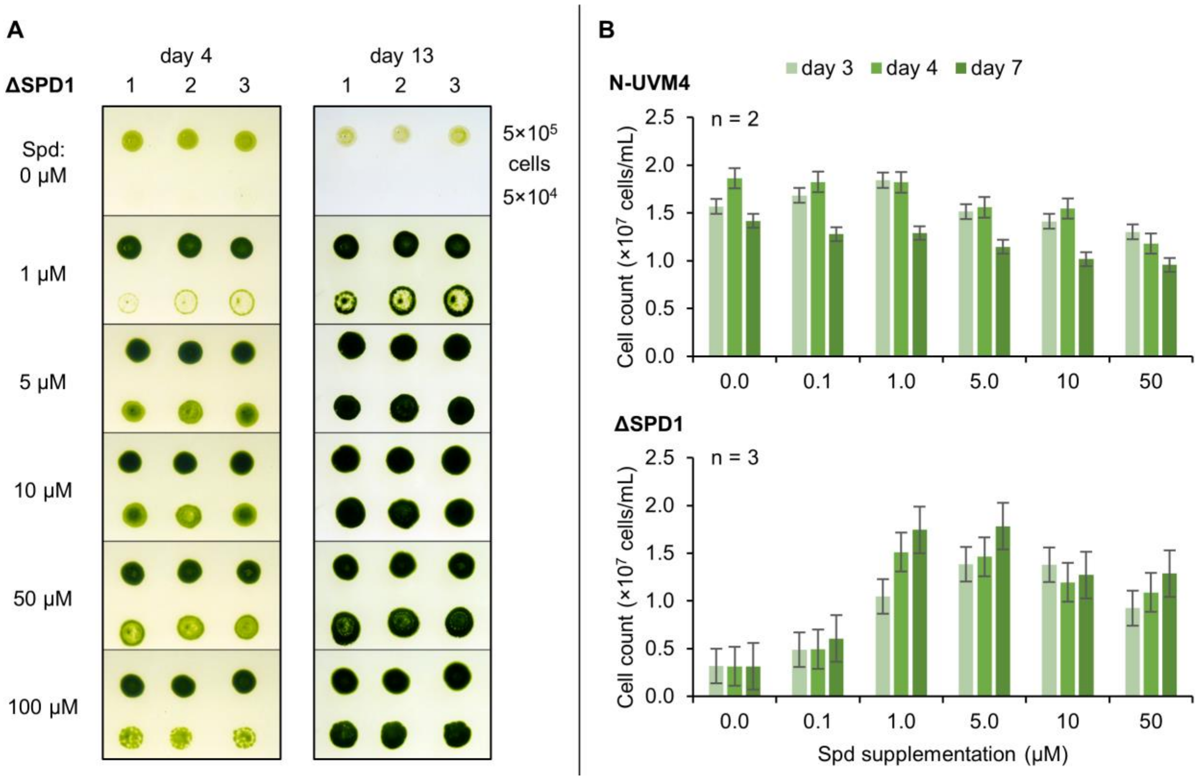

Figure S8: Spermidine supplementation requirements of the ΔSPD1 mutant. (**A**) Growth of three ΔSPD1 mutants on solid TAP medium containing indicated amounts of spermidine. 20 µL of cell suspension containing a total of about 500.000 and 50.000 cells respectively was spotted onto the plates and pictures were taken four and 13 days later. (**B**) Cell concentration of the N-UVM4 parental strain (top) and three ΔSPD1 mutants (bottom) in liquid TAP medium containing indicated amounts of spermidine three, four and seven days after inoculation. Error bars represent the standard deviation of biological replicates as indicated. (Spd: spermidine)

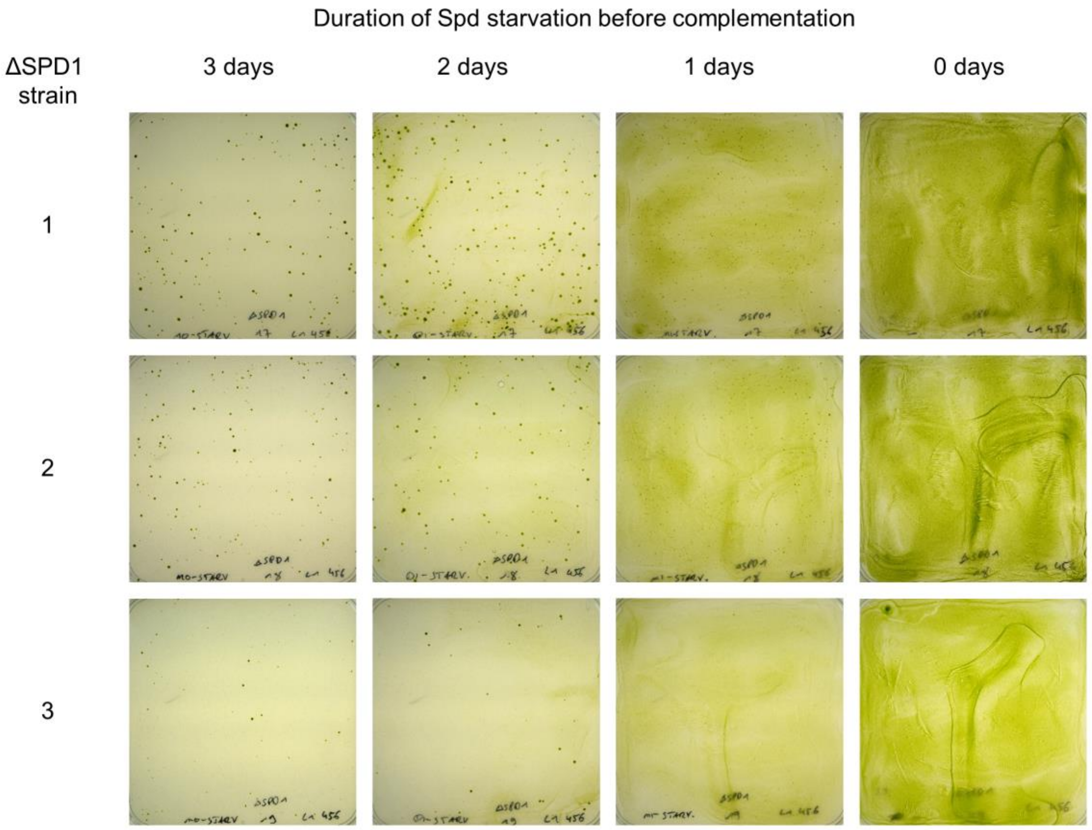

Figure S9: Effect of spermidine starvation prior to complementation of the ΔSPD1 mutation. Cells were transferred to spermidine-less medium and kept in exponential growth phase for the indicated amount of days prior to transformation with construct II. Pictures were taken five days after plating.

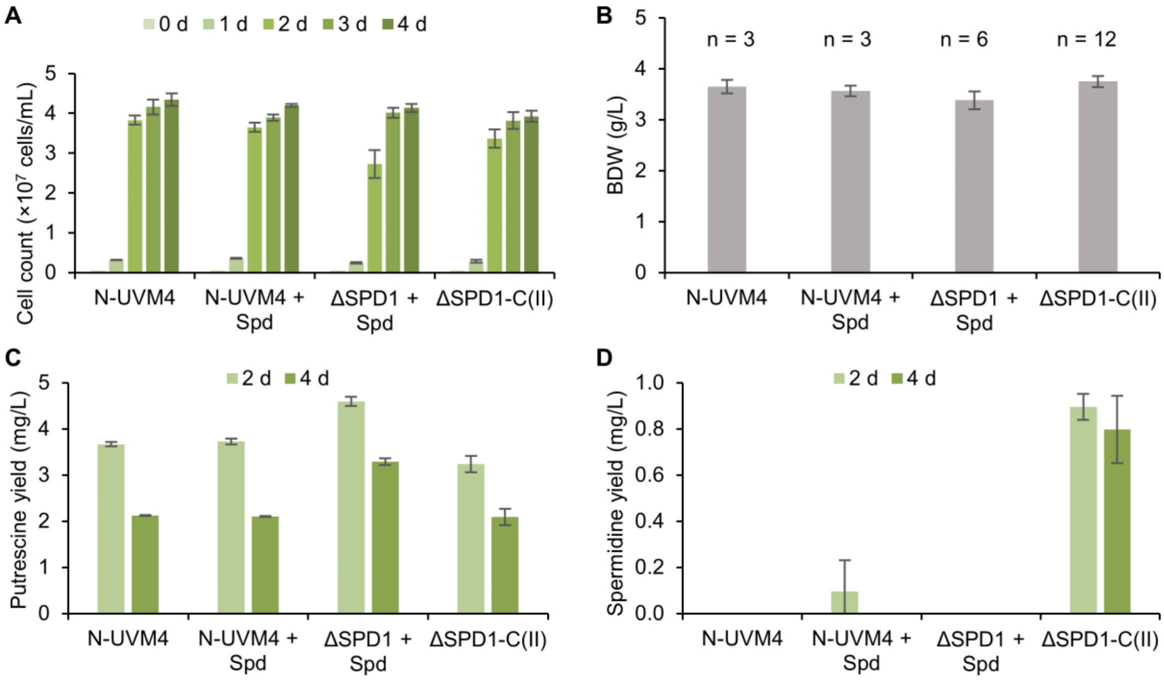

Figure S10: Phototrophic growth performance of six ΔSPD1 mutant strains compared to complemented and parental strains in T2P with CO_2 gassing. Three *SPD1* knockout strains were complemented using construct II, of which four progenitor strains were cultivated each. (**A**) Cell concentration over the course of the cultivation. (**B**) Biomass dry weight at day four. (**C**) Volumetric putrescine (left) and spermidine (right) yields of the cellular fraction at day two and four. For missing columns, yields were below the limit of detection. Error bars represent the standard deviation of indicated biological replicates.

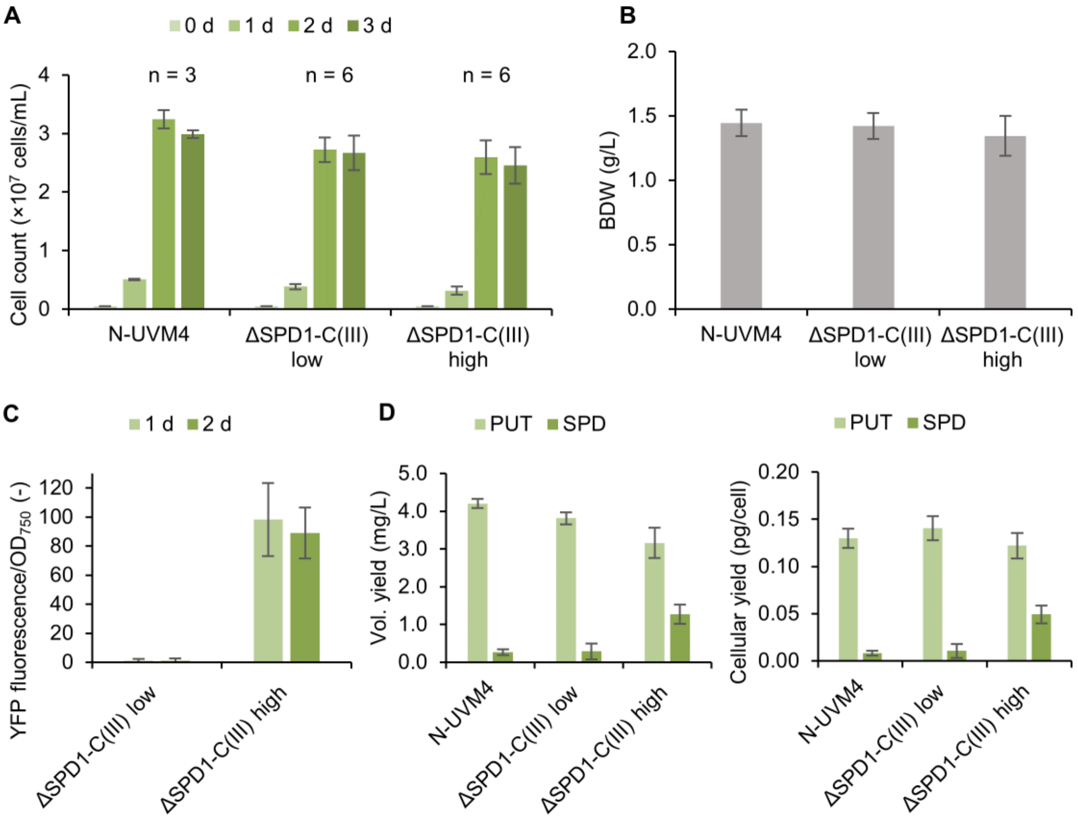

Figure S11: Mixotrophic growth performance of strains with low and high levels of SPD1-YFP expression compared to the strain N-UVM4. Three *SPD1* knockout strains were complemented using construct III, of which two progenitor strains were cultivated each. (**A**) Cell concentration over the course of the cultivation. (**B**) Biomass dry weight at day three. (**C**) OD-based YFP fluorescence levels at day one and two. (**D**) Volumetric (left) and cellular (right) putrescine and spermidine yields of the cellular fraction at day two. Error bars represent the standard deviation of indicated biological replicates.
